# A comparison of gender-linked population cancer risks between alcohol and tobacco: how many cigarettes are there in a bottle of wine?

**DOI:** 10.1186/s12889-019-6576-9

**Published:** 2019-03-28

**Authors:** Theresa J. Hydes, Robyn Burton, Hazel Inskip, Mark A. Bellis, Nick Sheron

**Affiliations:** 1 Department of Gastroenterology and Hepatology, University Hospital Southampton NHS Foundation Trust, Tremona Road, Southampton, SO16 6YD UK; 2 Public Health England, Wellington House, 133-155 Waterloo Road, London, SE1 8UG UK; 3 MRC Lifecourse Epidemiology Unit, University of Southampton, University Hospital Southampton NHS Foundation Trust Tremona Road, Southampton, SO16 6YD UK; 4 NIHR Southampton Biomedical Research Centre, University of Southampton, University Hospital Southampton NHS Foundation Trust Tremona Road, Southampton, SO16 6YD UK; 5 College of Health and Behavioural Sciences, Bangor University, Bangor, LL57 2UW UK; 6 Faculty of Medicine, University of Southampton, Mailpoint 81, Level E, South Academic Block, University Hospital Southampton NHS FoundationTrust, Tremona Road, Southampton, SO16 6YD UK; 7 World Health Organization Collaborating Centre on Investment in Health and Well-being, Public Health Wales, Cardiff, CF10 4BZ UK

**Keywords:** Alcohol, Smoking, Tobacco, Cancer, Breast cancer

## Abstract

**Background:**

In contrast to our knowledge about the number of cancers attributed to smoking, the number of cancers attributed to alcohol is poorly understood by the public. We estimate the increase in absolute risk of cancer (number of cases per 1000) attributed to moderate levels of alcohol, and compare these to the absolute risk of cancer attributed to low levels of smoking, creating a ‘cigarette-equivalent of population cancer harm’.

**Methods:**

Alcohol and tobacco attributable fractions were subtracted from lifetime general population risks of developing alcohol- and smoking-related cancers, to estimate the lifetime cancer risk in alcohol-abstaining non-smokers. This was multiplied by the relative risk of drinking ten units of alcohol or smoking ten cigarettes per week, and increasing levels of consumption.

**Results:**

One bottle of wine per week is associated with an increased absolute lifetime cancer risk for non-smokers of 1.0% (men) and 1.4% (women). The overall absolute increase in cancer risk for one bottle of wine per week equals that of five (men) or ten cigarettes per week (women). Gender differences result from levels of moderate drinking leading to a 0.8% absolute risk of breast cancer in female non-smokers.

**Conclusions:**

One bottle of wine per week is associated with an increased absolute lifetime risk of alcohol-related cancers in women, driven by breast cancer, equivalent to the increased absolute cancer risk associated with ten cigarettes per week. These findings can help communicate that moderate levels of drinking are an important public health risk for women. The risks for men, equivalent to five cigarettes per week, are also of note.

**Electronic supplementary material:**

The online version of this article (10.1186/s12889-019-6576-9) contains supplementary material, which is available to authorized users.

## Background

The health risks of smoking are indisputable and well understood by the public. Tobacco use accounts for 7 million deaths per year globally with an estimated two thirds of smokers expected to die from their habit [1, 2]. Smoking is responsible for 22% of cancer deaths worldwide [3]. Heavy taxes, advertising bans, plain packaging containing explicit health warnings, and a ban on smoking in public places has led to a decrease in smoking prevalence from 46% (1974) to 19% (2014) [4], and over 70% of the population now understand smoking to be a major cause of cancer compared with 40% in 1966 [5]. For many years the tobacco industry tried to suppress information linking tobacco use and cancer [6], and there is evidence that the alcohol industry are currently employing similar tactics [7, 8].

Each year approximately 3.3 million deaths occur due to the harmful use of alcohol, corresponding to 5.9% of all deaths globally [9]. Furthermore alcohol was the leading cause of death among 15–49 year olds worldwide in 2016 [10]. It is estimated that alcohol use lead to 167,000 years of working life lost (YWLL) in England in 2015, 16% of the total and more than that for the ten most frequent cancers combined [11]. However alcohol is generally perceived as being comparatively less harmful by the public, particularly in terms of cancer, despite being directly linked to carcinoma of the oropharynx, larynx, oesophagus, colorectum, liver and breast [12–14]. In a recent survey of 2100 adults only 13% named cancer as a health risk of hazardous drinking, and when presented with a list of alcohol-related cancers only 18 and 40% correctly identified breast and colorectal cancer respectively, despite alcohol being linked to 3200 and 4800 cases of each per year in the United Kingdom (UK) [15].

All known alcohol-related cancers have been shown to have a modest, but increased relative risk (RR) of incidence at moderate levels of drinking (Additional file 1: Table S1) [13, 14, 16]. There is now robust evidence that low levels of alcohol intake do not provide any protective health benefits [10], and The World Health Organisation (WHO) International Agency for Research on Cancer (IARC), World Cancer Research Fund and American Institute for Cancer Research have all stated that no level of alcohol consumption is completely safe [12, 17–19]. This led to recent reform of the 1995 UK sensible drinking guidelines to state that any level of alcohol consumption can be associated with range of cancers and there is no justification for drinking for health reasons [20].

The aim of this study was to estimate the increase in absolute risk of developing cancer as a result of drinking moderate levels of alcohol in men and women, and compare this to the increase in absolute risk of developing cancer secondary to smoking. In essence, we aim to answer the question: ‘Purely in terms of cancer risk - how many cigarettes are there in a bottle of wine’?

## Methods

**Fig. 1:**
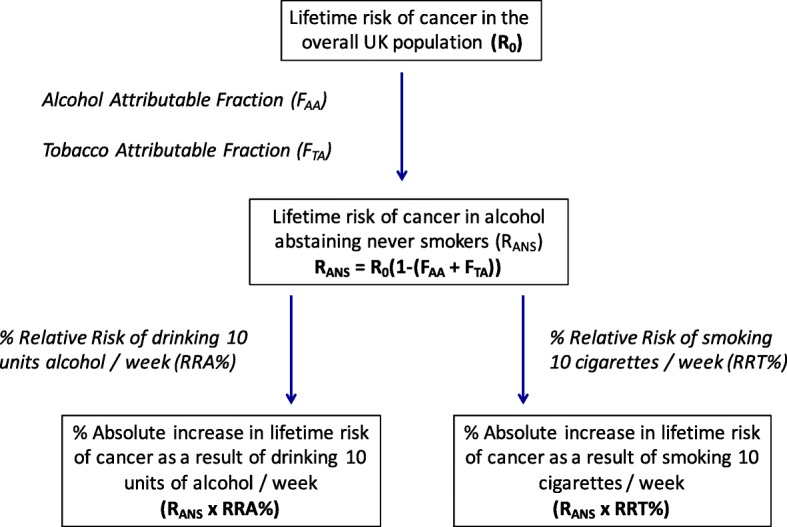
Methodology used to calculate the percentage absolute increase in lifetime risk of cancer attributable to drinking ten units of alcohol, or smoking ten cigarettes per week. * Fig. 1 illustrates the percentage increase in RR of cancer due to consuming ten units of alcohol or ten cigarettes per week, however RR for consuming higher quantities of alcohol and tobacco were also examined

In order to calculate the absolute increase in lifetime cancer risk (AR) resulting from consumption of ten units of alcohol (one bottle of wine) or ten cigarettes per week we began by subtracting the sum of the Alcohol Attributable Fraction (F_AA_) (Alcohol Attributable Fraction for England 2013) [21] for all known alcohol-related cancers and Tobacco Attributable Fractions (F_TA_) (Tobacco-attributable cancer burden in the UK) [22] for all tobacco-related cancers from the overall lifetime risk (R_0_) of cancer (Cancer Research UK (CRUK) 2010) [23] for the general population (Additional file 1: Table S2). This allowed us to calculate the lifetime risk of cancer in alcohol-abstaining never smokers (R_ANS_), according to the expression: R_ANS_ = R_0_ – R_0_F_AA_ – R_0_F_TA,_ or alternatively: R_ANS_ = R_0_(1 – (F_AA_ + F_TA_)) (Fig. 1). Lifetime cancer risk calculations were performed by the CRUK Cancer Intelligence Team based on data provided by the Office for National Statistics, ISD Scotland, Welsh Cancer Intelligence and Surveillance Unit, and the Northern Ireland Cancer Registry, (December 2013 to July 2014). The methodology used is described by Sasieni et al. [24].

There is well-established synergy between alcohol and tobacco consumption for oropharyngeal and gastrointestinal (GI) tract cancer [25, 26]. Therefore the sum of the F_AA_ (calculated from a population of smokers and non-smokers) [21], and F_TA_ (calculated from a population of drinkers and non-drinkers) [22], would over-estimate their combined contribution towards cancer risk. We therefore adjusted for this by multiplying the F_AA_ by the frequency of alcohol-consumers who are never-smokers (53%), and the F_TA_ by the frequency of smokers who do not consume alcohol (12%), for cancers in which both alcohol and tobacco are known risk factors (Additional file 1: Table S2). These data were taken from the Health Survey for England data for the years 2011–14 (Public Health England) (Additional file 1: Table S3) [27].

We then multiplied the lifetime risk of cancer in never-smoking non-drinkers by the percentage increase in RR (calculated as: (RR-1)*100) of drinking ten (approximately one bottle of wine) and 30 (approximately three bottles of wine) units of alcohol per week (Alcohol Attributable Fraction for England 2008) [28] (Additional file 1: Table S1) to estimate the increase in absolute lifetime risk (expressed as a percentage) of developing cancer for this level of consumption in non-smokers (Fig. 1, Additional file 1: Table S4). The same method was used to calculate the percentage increase in absolute lifetime risk of smoking ten or 30 cigarettes per week in non-drinkers (Additional file 1: Table S4).

RR data for the consumption of ten units of alcohol per week was derived from studies which had adjusted for smoking in the case of upper aerodigestive tract cancers [13]. RR data for smoking was taken from studies which had adjusted for alcohol [29, 30], or excluded either daily drinkers [31], or all drinkers [32]. The percentage RR of smoking ten cigarettes per week were calculated using figures from the largest and most recent meta-analyses, case-control, or cohort studies available describing the risk of smoking according to frequency of cigarettes smoked, and where the primary end point was cancer incidence (Additional file 1: Table S5). Starting with the risk of smoking approximately five cigarettes per day (35 cigarettes per week), generally the lowest level of risk detailed in these studies, we estimated the RR of smoking just ten cigarettes per week using a log transformation (Additional file 1: Table S5, Additional File 2).

It is generally assumed smokers are dependent daily users, with tobacco consumption measured in cigarettes per day, despite an increasing prevalence of non-daily smokers in western countries [33]. However in non-dependent drinkers, alcohol consumption varies day-to-day and weekly consumption is the normal metric, one UK unit = 1 cl (8 g alcohol). In this study we discuss relatively low levels of alcohol and cigarette consumption and for ease of comparison we use weekly measures familiar to the general public for both throughout, and estimate a bottle of wine contains ten units = 10 cl (80 g alcohol). In an analysis of data from the Health Survey for England, the mean alcohol intake in hazardous drinkers was around 27 units per week; so as a further comparator we use three bottles of wine, or 30 units per week [34].

Some of the relative risk data from epidemiology studies are heterogeneous, and as a result any calculation must be inexact to some extent, and attempts to calculate confidence intervals are likely to give a misleading impression of precision. We therefore performed a sensitivity analysis reducing F_AA_, F_TA_ and the calculated RR of drinking ten units of alcohol, or smoking ten cigarettes per week, by a factor of 50% for cancers in which both alcohol and tobacco contribute (Additional file 1: Table S6).

## Results

### Absolute lifetime risk of cancer due to drinking one bottle of wine per week in non-smokers

**Fig. 2:**
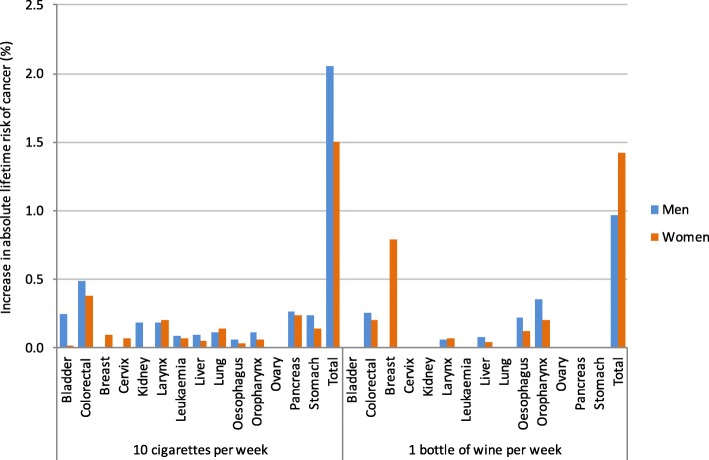
Comparison of the percentage increase in absolute lifetime risk of alcohol and tobacco-related cancers due to smoking ten cigarettes (in alcohol abstainers) or drinking ten units of alcohol (in non-smokers) per week

In non-smoking men the increase in the absolute lifetime risk of cancer from drinking one bottle of wine per week was 1.0%. For non-smoking women this was approximately 50% higher with an increase in absolute cancer risk of 1.4% (Additional file 1: Table S4, Fig. 2). In men this risk is due to cancers of the GI tract (oropharynx, oesophageal, colorectal, liver), whereas in women breast cancer accounts for 55% of additional cases. This is relevant because smoking is also an important cause of GI tract cancer, but not breast cancer. If 1000 men and 1000 women each drank one bottle of wine per week, we estimate around ten men and 14 women would develop cancer as a result.

A direct comparison of the percentage increase in absolute lifetime risk of all alcohol and tobacco-related cancers for smoking ten cigarettes per week, or drinking ten units of alcohol per week revealed that low levels of smoking carried the greatest risk for men (2.1% AR per ten cigarettes, 1.0% AR per ten units of alcohol), with the risk spread throughout the smoking-related cancers. The increase in absolute lifetime cancer risk for consuming ten cigarettes per week compared with ten units of alcohol for women however was comparable (1.5% AR per ten cigarettes, 1.4% AR per ten units of alcohol) as a result of breast cancer incidence being partly driven by alcohol (Additional file 1: Table S4).

**Fig. 3:**
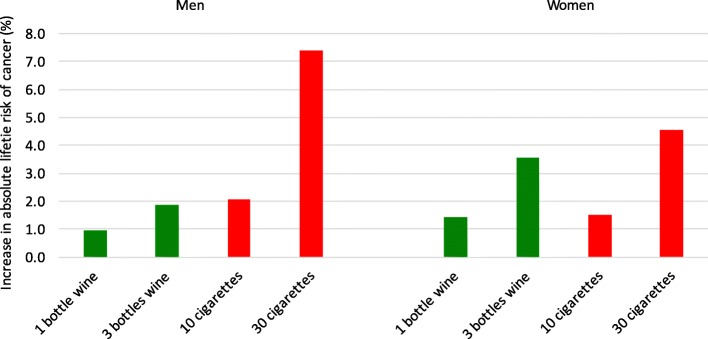
Comparison of the percentage increase in absolute lifetime risk of alcohol and tobacco-related cancers due to increasing levels of smoking and alcohol consumption per week

As alcohol consumption increases, differences in absolute lifetime risk of alcohol-related cancer between men and women become elevated. Drinking three bottles of wine per week (approximately half a bottle per day), is associated with in absolute lifetime cancer risk to 1.9% for men and 3.6% for women, again due to a significant risk of breast cancer (2.4%) (Additional file 1: Table S4, Fig. 3). Therefore if 1000 male and 1000 female non-smokers drink three bottles of wine per week throughout their lives, around 19 men and 36 women may develop an alcohol-related cancer as a result. The increase in absolute lifetime risks of cancer due to smoking are greater for men compared to women, for nearly all non-female cancers (Additional file 1: Table S4). As breast cancer is the most commonly occurring cancer for women, and is attributable to alcohol and not smoking, a gender gap in smoking-related absolute cancer risk can be seen in alcohol abstainers, which widens with increasing levels of tobacco exposure (Fig. 2 and Fig. 3, Appendix).

### In terms of cancer risk, how many cigarettes are there in a bottle of wine?

The increase in absolute cancer risk of drinking one bottle of wine per week is roughly equivalent to five cigarettes per week (4.7) for men ((1.0/2.1) × 10), and ten cigarettes (9.5) for women ((1.4/1.5) × 10). Drinking three bottles of wine a week, a representative consumption in hazardous drinkers, carries the same increase in absolute cancer risk of smoking roughly eight cigarettes per week for men, and 23 per week for women (approximately one packet) (Fig. 3).

### Sensitivity analysis

Sensitivity analysis adjustments led to a reduction in all absolute lifetime cancer risks (men: 1.7% AR per ten cigarettes, 0.6% AR per ten units alcohol; women: 1.2% AR per ten cigarettes, 1.2% AR per ten units alcohol). Consumption of ten units of alcohol per week carried an equivalent increase in absolute cancer risk to four cigarettes per week for men (3.5), and ten cigarettes per week for women (9.8), as breast cancer risks did not require adjustment.

## Discussion

We must first be absolutely clear that this study is not saying that drinking alcohol in moderation is in any way equivalent to smoking. Smoking kills up to two thirds of its users [2], and cancer is just one of the many serious health consequences. This study purely addresses cancer risk in isolation. The UK chief medical officer’s moderate drinking guideline of 14 units per week is set at a level at which there is a 1 % absolute risk of mortality from alcohol [35]. Furthermore the average consumption of cigarettes by smokers is around 80 per week in the UK and 100 per week in the United States (US) [36], far greater than our cigarette equivalent for moderate drinkers.

Using previously published resources reporting lifetime cancer risk, alcohol and tobacco attributable fractions and relative risk data for low to moderate levels of drinking and smoking, we have been able to estimate the approximate absolute lifetime risk of cancer in a non-smoking population, associated with moderate levels of alcohol consumption, and derive a cigarette equivalent in terms of harm. The ‘cigarette equivalent’ of a bottle of wine is five cigarettes for men and ten for women. The absolute risk of cancer increases with increasing alcohol consumption and the gender gap widens as a result of the association between alcohol and breast cancer. These figures occur on a background of a steady increase in alcohol consumption among women [37]. The latest survey estimates of alcohol consumption for 16–75 year old women in England is 1.4 units per day (ten units per week) [21]. Survey data underestimate intake, partly due to variations of units per glass of wine or pint of beer, and account for only 50% of alcohol consumption as measured by Her Majesty’s Revenue and Customs (HMRC) duty receipts. On this basis the average alcohol consumption for female drinkers is at least ten units per week. Given the approximate number of women in the UK aged 16–75 is 23,809,000 [38], current average levels of alcohol intake could result in around 339,000 extra cancers. It is also worth considering that there is a higher incidence of smoking among social drinkers compared to alcohol abstainers [39]. This is important as smoking can substantially increase the carcinogenic risk associated with alcohol consumption, particularly for cancers of the upper aerodigestive tract [25, 26].

While the association between hazardous drinking and breast cancer is well established [12, 14], a number of studies have highlighted a risk with low to moderate levels of alcohol consumption [14, 16, 40, 41]. A pooled analysis from 53 studies recruiting 58,515 women with breast cancer was the first to reveal that moderate levels of alcohol are associated with increased RR of developing breast cancer independent of smoking [14]. The UK’s Million Women Study reported an excess incidence of 15 per 1000 cancer cases for each additional alcoholic drink consumed per day, 11 due to breast cancer [14, 16]. These figures are comparable to our findings of 14 extra cancers per 1000 women drinking ten units per week (eight due to breast cancer) in non-smokers. The latest results from the European Prospective Investigation into Cancer and Nutrition, found an elevated hazard ratio for developing breast cancer of 4.2% for every 10 g (1.25 units) of alcohol consumed per day [40]. More recently Cao et al. presented data from the Nurses’ Health Study and Health Professionals Follow-up Study and demonstrated just one alcoholic drink per day could increase the RR of alcohol-related cancers independent of smoking [41]. Again this was driven by breast cancer and restricted to women [41]. While these studies support our data, our study is the first description of a percentage increase in absolute lifetime risk of cancer related to alcohol and the only study to provide a ‘cigarette equivalent’ of harm.

Rising levels of alcohol consumption over the past decade, are likely to be an important factor in the over 30% rise in breast cancer incidence in England (36,509 registrations in 2003, 55,122 in 2015) [42]. Breast cancer is now the most common cancer in UK women [43]. Exceptional efforts have been made to improve survival, however 11,563 women still died from breast cancer in the UK in 2016 and treatment carries a huge cost burden [42]. The benefits of increasing public awareness of the risks of moderate drinking could therefore be immense. It is important to educate women that an increased risk of breast cancer is not restricted to hazardous and harmful drinkers, and the messaging needs to be simple, relevant and memorable. Furthermore we hope this data will inform Chief Medical Officers of further opportunities for prevention of cancer at a population level.

It is interesting to consider that the list of alcohol-related cancers selected by the WHO IARC may be conservative, as several other cancers have recently been identified as having a dose-risk relationship to alcohol including melanoma, cancer of the gallbladder, pancreas, lung and prostate [44, 45]. Furthermore where papers have controlled for the ‘sick-quitter’ effect, alcohol-related RRs of developing cancer are substantially higher [45]. These two factors suggest that the cancer risk associated with moderate levels of drinking described in this paper may be underestimated for both men and women, although the gender gap may be smaller.

There are caveats with the methodology used in the study. Ideally all our risk estimates would have been calculated from systematic reviews and meta-analyses of alcohol consumption in non-smoking men and women, and cigarette consumption in teetotal men and women. While these data are available in a few individual studies, systematic review data are not available for subgroups of cancer type.

We have therefore estimated the risks for each cancer type using the most recent and robust available data and by making a number of assumptions. The data on RRs for cancer are extremely heterogenous, and while the original studies controlled for the use of other substances, our methodology does not account for synergies between RRs of alcohol and tobacco. In order to remove some of the confounding influence where these two risk factors co-exist, we adjusted the F_AA_ and F_TA_ to include non-smokers and non-drinkers only in alcohol-related and smoking-related cancers respectively and used adjusted RR data. We acknowledge however that the alcohol history of smokers is generally less accurate, and that a higher proportion of smokers may have previously been heavy drinkers. This factor may have led to a minor underestimate of the combined cancer risk. Furthermore F_AA_ and alcohol-related RRs for cancer are derived largely from mortality, rather than morbidity data, and are therefore both likely to be underestimated, particularly in the case of breast cancer where the ratio of incidence to mortality is high (Additional file 1: Table S7) [23]. Therefore, while we were unable to calculate confidence intervals on our estimates we have tried to be conservative with our assumptions.

The best data available to us on the RR of cancer attributable to moderate levels of alcohol (ten units per week) were for consumption of less than 19 g per day, an average of 8.8 units per week if normally distributed. Similarly, our estimate of 30 units per week was derived from individuals drinking 20-39 g per day, i.e. 25.9 units per week (Additional file 1: Table S1) [28]. These data would slightly underestimate our calculation of the absolute increase in cancer risk associated with drinking ten and 30 units per week. In terms of tobacco, there is a lack of published data on RRs of cancer attributable to very low levels of smoking, with the lowest level of exposure generally reported as one to ten (approximately five) cigarettes per day. While recent studies have looked at lower levels of smoking their end point is mortality, not cancer incidence [46, 47]. While we were therefore required to estimate the risks of developing cancer at lower levels of consumption (just ten cigarettes per week) using logarithmic transformation, we believe this is justified as exposure to any level of carcinogens within cigarette smoke is likely to cause harm, and there is no reason to suppose there is a threshold at which this risk would begin [48]. As a result of these uncertainties we performed a sensitivity analysis reducing F_AA_, F_TA_ and RRs for consuming ten units alcohol per week or ten cigarettes per week by 50%. This reduced the ‘cigarette equivalent’ of one bottle of wine to four for men but remained ten for women.

This study does not account for the duration an individual is exposed to a cancer-related risk factor. Cumulative exposure is directly related to the incidence of certain cancers, particularly tobacco smoking and lung cancer [49]. Furthermore this study only takes into account cancer incidence and not the age at which a cancer develops and its prognosis. However while the majority of alcohol-related cancers, including breast cancer may have a better ten year survival than lung cancer (78% vs 5%, Additional file 1: Table S7), the years of life lost from cancer are comparable. Lung cancer is the leading cause of years of life lost from cancer (2,365,000 years of life lost), but colorectal cancer is the second (804,000 years of life lost), and female breast cancer the third (778,000 years of life lost) [50]. In addition breast cancer affects women at a relatively younger age compared to lung cancer [51], resulting in a significant burden for them and their families, including young children.

It is noteworthy that the attributable fractions and the absolute risk scores calculated here are dependent on both the prevalence of exposure to a risk factor within a population and the relative risk of that behaviour resulting in cancer. For smoking the prevalence is low (approximately 20% of individuals in the UK are daily smokers for example [52]), with a high relative risk of cancer for current smokers; whereas alcohol consumption is more common (72% of women and 83% of men consume some alcohol in countries with a high socio-demographic index [10]), but is associated with a lower relative risk of cancers overall; even for high levels of consumption [53]. Critically, our findings are not meant to detract from the substantive cancer risks associated with smoking which remains the single largest preventable cause of cancer worldwide [54], and for which even very low levels of exposure are associated with an increased risk of cancer [46].

Critically our use of absolute ‘lifetime risk’ describes the average of the number of cancers experienced by a population, i.e. individuals with and without cancer, and is not an exact measure of an individual’s probability of getting cancer. In other words while this study estimates that drinking ten units of alcohol per week may cause a similar number of cancers in the population as smoking five to ten cigarettes per week, these two exposures may not carry the same cancer risk for any given individual. We have attempted to minimise this effect by using lifetime cancer risks from CRUK which have been calculated using the Sasieni method. This corrects incidence rates for the inclusion of more than one primary cancer occurring within the same individual, lowering the lifetime cancer risk [24].

Furthermore this study does not take into account other smoking or alcohol-related outcomes such as respiratory, cardiovascular or liver disease in which case the conclusions would likely be quite different. Cancer deaths are a fraction of the total number of deaths associated with smoking and alcohol and this study is not a comparison of the overall mortality of smoking versus alcohol. Despite the caveats, our estimation of a ‘cigarette equivalent’ for alcohol provides a useful measure for communicating cancer risks that exploits successful historical messaging on smoking, reflects current epidemiological knowledge and includes an important aspect of gender differential.

## Conclusions

We have shown moderate levels of drinking (one bottle of wine per week) is associated with a significant increased absolute lifetime risk of alcohol-related cancers in women, driven largely by breast cancer. Drinking one bottle of wine per week is associated with an increase in absolute lifetime risk of cancer equivalent to smoking ten cigarettes a week for women, and five for men. These findings highlight moderate levels of drinking as an important public health issue for women and identify a need to promote national awareness, supported by the recent change in national drinking guidelines. This study offers the first attempt to use well established and well communicated links between cancer and tobacco as a mechanism to explain similar links between alcohol and cancer which have to date failed to reach most of the public.

## Additional files

Additional file 1: **Table S1.** References for the percentage relative risk of drinking ten units of alcohol per week. **Table S2.** Calculation of lifetime risk of cancer in alcohol abstaining never smokers. **Table S3.** Frequency of smoking and alcohol consumption in the UK, Health Survey for England data combined years 2011–14 (Public Health England n.d.). **Table S4.** Calculation of absolute increase lifetime risk of cancer due to ten and 30 units of alcohol or ten and 30 cigarettes per week in non-smokers and non-drinkers respectively. **Table S5.** References and calculation of absolute increase lifetime risk of cancer due to ten and 30 cigarettes per week. **Table S6.** Calculation of absolute increase lifetime risk of cancer due to ten units of alcohol or ten cigarettes per week in non-smokers and non-drinkers respectively following a sensitivity analysis. **Table S7.** Incidence and mortality data for alcohol-related cancers (CRUK data). (DOCX 76 kb)

Additional file 2: Contains a worked example of logarithmic transformation used to calculate RR of smoking 10 cigarettes per week. (DOCX 31 kb)

## Abbreviations

AR: Absolute increase in lifetime cancer risk
CRUK: Cancer Research United Kingdom
GI: Gastrointestinal
HMRC: Her Majesty’s Revenue and Customs
IARC: International Agency for Research on Cancer
RR: Relative Risk
UK: United Kingdom
US: United States
WHO: World Health Organisation
YWLL: Years of working life lost

## References

[1] Doll R, Peto R, Wheatley K, Gray R, Sutherland I (1994). Mortality in relation to smoking: 40 years’ observations on male British doctors. BMJ..

[2] World Health Organisation. Fact Sheets. Tobacco.

[3] Forouzanfar MH, Afshin A, Alexander LT, Anderson HR, Bhutta ZA, Biryukov S (2016). Global, regional, and national comparative risk assessment of 79 behavioural, environmental and occupational, and metabolic risks or clusters of risks, 1990–2015: a systematic analysis for the global burden of disease study 2015. Lancet..

[4] Health and Social Care Information Centre. Statistics on Smoking, England, 2016. 2016.

[5] Saad L (2002). Tobacco and smoking [internet].

[6] Proctor RN (2012). The history of the discovery of the cigarette-lung cancer link: evidentiary traditions, corporate denial, global toll. Tob Control.

[7] Petticrew M, Maani Hessari N, Knai C, Weiderpass E. How alcohol industry organisations mislead the public about alcohol and cancer. Drug Alcohol Rev. 2017. 10.1111/dar.12596. 10.1111/dar.12596 28881410

[8] Picard A. Removing warning labels from Yukon liquor is shameful. The Globe and Mail. 2018.

[9] World Health Organisation. Global Status Report on non communicable diseases 2014. 2014.

[10] Global Burden of Disease Alcohol Collaborators. Alcohol use and burden for 195 countries and territories, 1990–2016: a systematic analysis for the Global Burden of Disease Study 2016. Lancet. 2018;0(0). 10.1016/S0140-6736(18)31310-2 PMC6148333 30146330

[11] Burton R, Henn C, Lavoie D, O’Connor R, Perkins C, Sweeney K (2017). A rapid evidence review of the effectiveness and cost-effectiveness of alcohol control policies: an English perspective. Lancet..

[12] Baan R, Straif K, Grosse Y, Secretan B, El Ghissassi F, Bouvard V (2007). Carcinogenicity of alcoholic beverages. Lancet Oncol..

[13] Corrao G, Bagnardi V, Zambon A, La Vecchia C. A meta-analysis of alcohol consumption and the risk of 15 diseases. Prev Med (Baltim). 2004 May [cited 2018 May 27];38(5):613–619. 10.1016/j.ypmed.2003.11.027 15066364

[14] Hamajima N, Hirose K, Tajima K, Rohan T, Calle EE, Heath CW (2002). Alcohol, tobacco and breast cancer--collaborative reanalysis of individual data from 53 epidemiological studies, including 58,515 women with breast cancer and 95,067 women without the disease. Br J Cancer.

[15] Buykx P, Li J, Gavens L, Lovatt M, Gornes de Matos E, Holmes J, et al. Public awareness of the link between alcohol and cancer in England in 2015: a population-based survey. BMC Public Health. 2016:16–1194. 10.1186/s12889-016-3855-6 PMC5129195 27899099

[16] Allen NE, Beral V, Casabonne D, Kan SW, Reeves GK, Brown A (2009). Moderate alcohol intake and cancer incidence in women. J Natl Cancer Inst.

[17] Latino-Martel P, Arwidson P, Ancellin R, Druesne-Pecollo N, Hercberg S, Le Quellec-Nathan M (2011). Alcohol consumption and cancer risk: revisiting guidelines for sensible drinking. CMAJ..

[18] Secretan B, Straif K, Baan R, Grosse Y, El Ghissassi F, Bouvard V (2009). A review of human carcinogens-part E: tobacco, areca nut, alcohol, coal smoke, and salted fish. Lancet Oncol.

[19] World Cancer Research Fund/American Institute for Cancer Research. Food, nutrition physical activity and the prevention of cancer: a global perspective. Washington, USA; 2007.

[20] Alcohol Policy Team Department of Health. How to keep health risks from drinking alcohol to a low level: Government response to the public consultation. 2016.

[21] Jones L, Bellis M (2014). Updating England-specific alcohol-attributable fractions for England 2013.

[22] Parkin DM (2011). Tobacco-attributable cancer burden in the UK in 2010. Br J Cancer.

[23] Cancer Research UK (2010). Statistics.

[24] Sasieni PD, Shelton J, Ormiston-Smith N, Thomson CS, Silcocks PB (2011). What is the lifetime risk of developing cancer?: the effect of adjusting for multiple primaries. Br J Cancer.

[25] Prabhu A, Obi KO, Rubenstein JH (2014). The synergistic effects of alcohol and tobacco consumption on the risk of esophageal squamous cell carcinoma: a meta-analysis. Am J Gastroenterol.

[26] Dal Maso L, Torelli N, Biancotto E, Di Maso M, Gini A, Franchin G (2016). Combined effect of tobacco smoking and alcohol drinking in the risk of head and neck cancers: a re-analysis of case-control studies using bi-dimensional spline models. Eur J Epidemiol.

[27] Public Health England. Analysis of Health Survey for England data combined years 2011–14 (unpubished data). 2017.

[28] Jones L, Bellis M, Deadman D, Sumnall H, Tocque K (2008). Alcohol-attributable fractions for England 2008.

[29] Ordóñez-Mena JM, Schöttker B, Mons U, Jenab M, Freisling H, Bueno-de-Mesquita B (2016). Quantification of the smoking-associated cancer risk with rate advancement periods: meta-analysis of individual participant data from cohorts of the CHANCES consortium. BMC Med.

[30] Lagergren J, Bergström R, Lindgren A, Nyrén O (2000). The role of tobacco, snuff and alcohol use in the aetiology of cancer of the oesophagus and gastric cardia. Int J Cancer.

[31] Koh W-P, Robien K, Wang R, Govindarajan S, Yuan J-M, Yu MC (2011). Smoking as an independent risk factor for hepatocellular carcinoma: the Singapore Chinese health study. Br J Cancer.

[32] Hashibe M, Brennan P, Benhamou S, Castellsague X, Chen C, Curado MP (2007). Alcohol drinking in never users of tobacco, cigarette smoking in never drinkers, and the risk of head and neck cancer: pooled analysis in the international head and neck Cancer epidemiology consortium. J Natl Cancer Inst.

[33] Schane RE, Glantz SA, Ling PM (2009). Nondaily and social smoking: an increasingly prevalent pattern. Arch Intern Med.

[34] Sheron N, Gilmore I (2016). Effect of policy, economics, and the changing alcohol marketplace on alcohol related deaths in England and Wales. BMJ..

[35] Department of Health. UK Chief Medical Officer Alcohol Guidelines, Summary of the proposed new guidelines. 2016.

[36] OECD Publishing. OECD statistics, non-medical determinants of health, tobacco consumption.

[37] World Health Organisation. Global status report on alcohol and health. 2014:2014.

[38] Office for National Statistics. 2011 Census: Population Estimates for the United Kingdom 2011.

[39] Johnson KA, Jennison KM (1992). The drinking-smoking syndrome and social context. Int J Addict.

[40] Romieu I, Scoccianti C, Chajès V, de Batlle J, Biessy C, Dossus L (2015). Alcohol intake and breast cancer in the European prospective investigation into cancer and nutrition. Int J Cancer.

[41] Cao Y, Willett WC, Rimm EB, Stampfer MJ, Giovannucci EL. Light to moderate intake of alcohol, drinking patterns, and risk of cancer: results from two prospective US cohort studies. BMJ. 2015 Jan;351:10.1136/bmj.h4238. 10.1136/bmj.h4238 PMC4540790 26286216

[42] Cancer Resesarch UK. Breast cancer statistics.

[43] Office for National Statistics. Registrations of cancer diagnosed in 2013, England. 2015.

[44] Bagnardi V, Rota M, Botteri E, Tramacere I, Islami F, Fedirko V, et al. Alcohol consumption and site-specific cancer risk: a comprehensive dose–response meta-analysis. Br J Cancer. 2014;112. 10.1038/bjc.2014.579 PMC4453639 25422909

[45] Zhao J, Stockwell T, Roemer A, Chikritzhs T (2016). Is alcohol consumption a risk factor for prostate cancer? A systematic review and meta–analysis. BMC Cancer.

[46] Bjartveit K, Tverdal A (2005). Health consequences of smoking 1-4 cigarettes per day. Tob Control.

[47] Inoue-Choi M, Liao LM, Reyes-Guzman C, Hartge P, Caporaso N, Freedman ND (2017). Association of Long-term, low-intensity smoking with all-cause and cause-specific mortality in the National Institutes of Health-AARP diet and health study. JAMA Intern Med.

[48] United States. Public Health Service. Office of the Surgeon General., United States. Office on Smoking and Health. The health consequences of smoking : a report of the Surgeon General. U.S. Dept. of Health and Human Services, Public Health Service, Office of the Surgeon General; 2004. 941 p.

[49] Peto R, Darby S, Deo H, Silcocks P, Whitley E, Doll R (2000). Smoking cessation, and lung cancer in the UK since 1950: combination of national statistics with two case-control studies. BMJ..

[50] National Cancer Institute. Person-Years of Life Lost | Cancer Trends Progress Report [Internet]. 2018.

[51] Cancer Resesarch UK. Breast and lung cancer incidence by age, UK 2013–15.

[52] Reitsma MB, Fullman N, Ng M, Salama JS, Abajobir A, Abate KH (2017). Smoking prevalence and attributable disease burden in 195 countries and territories, 1990–2015: a systematic analysis from the global burden of disease study 2015. Lancet.

[53] Brown KF, Rumgay H, Dunlop C, Ryan M, Quartly F, Cox A (2018). The fraction of cancer attributable to modifiable risk factors in England, Wales, Scotland, Northern Ireland, and the United Kingdom in 2015. Br J Cancer.

[54] World Health Organisation. International Agency for Research on Cancer. IARC monographs on the evaluation of carcinogenic risks to humans: Tobacco Smoke & Involunary Smoking. Tobacco Smoke & Involunary Smoking. Lyon, France; 2004.

