# Supplementary material for: A comparison of gender-linked population cancer risks between alcohol and tobacco: how many cigarettes are there in a bottle of wine?
Source: BMC Public Health. 2019 Mar 28;19:316. doi: 10.1186/s12889-019-6576-9 (PMC6437970; doi:10.1186/s12889-019-6576-9)
Supplement: Supplementary file 1 — Table S1. References for the percentage relative risk of drinking ten units of alcohol per week. Table S2. Calculation of lifetime risk of cancer in alcohol abstaining never smokers. Table S3. Frequency of smoking and alcohol consumption in the UK, Health Survey for England data combined years 2011–14 (Public Health England n.d.). Table S4. Calculation of absolute increase lifetime risk of cancer due to ten and 30 units of alcohol or ten and 30 cigarettes per week in non-smokers and non-drinkers respectively. Table S5. References and calculation of absolute increase lifetime risk of cancer due to ten and 30 cigarettes per week. Table S6. Calculation of absolute increase lifetime risk of cancer due to ten units of alcohol or ten cigarettes per week in non-smokers and non-drinkers respectively following a sensitivity analysis. Table S7. Incidence and mortality data for alcohol-related cancers (CRUK data). (DOCX 76 kb) [file 12889_2019_6576_MOESM1_ESM.docx]

**Supplementary Tables**

**Supplementary Table 1**. References for the percentage relative risk of drinking ten units of alcohol per week

|  |  |  |  |  | **Relative risk of consuming 1-19g/day alcohol**  **(approx. 10 units / week)*** | | **Relative risk of consuming 20-39g/day alcohol**  **(approx. 30 units / week)†** | |
| --- | --- | --- | --- | --- | --- | --- | --- | --- |
| Cancer type | Paper | Study | Size | No. cases | Men | Women | Men | Women |
| Colorectal | Corrao et al. *Prev Med.* 38(5):613–619 (2004) | Meta-analysis 156 studies (1966-1998) | 16 studies Colon  6 studies Rectum | 5360 Colon  1420 Rectum | 1.04  (1.03 colon,  1.05 rectum) | 1.04  (1.03 colon,  1.05 rectum) | 1.07  (1.05 colon,  1.06 rectum) | 1.07  (1.05 colon,  1.06 rectum) |
| Larynx |  |  | 20 studies | 3789 | 1.22 | 1.22 | 1.43 | 1.43 |
| Liver |  |  | 10 studies | 1321 | 1.10 | 1.10 | 1.19 | 1.19 |
| Oesophagus |  |  | 14 studies | 3233 | 1.20 | 1.20 | 1.39 | 1.39 |
| Oropharynx |  |  | 15 studies | 4507 | 1.43 | 1.43 | 1.86 | 1.86 |
| Breast | Hamajima et al*. Br J Cancer.* 87:1234-1245 (2002) | Reanalysis of 53 epidemiological studies | 153,582 subjects | 58,515 | - | 1.07 | - | 1.21 |

* 1-19g per day, i.e. 7-133g per week is the equivalent of 0.9-16.6 units per week (an approximate average of 10 units / week)

† 20-39g per day, i.e. 160-312g per week is the equivalent of 17.5-34.3 units per week (an approximate average of 30 units / week)

RR data was originally presented by Corrao et al for 25, 50, 100g alcohol per day, and Hamajima 0, <5, 5-14, 15-24, 25-34, 35-44, ≥45g alcohol per day. Estimates of the effects of 1-19g per day were calculated by Jones et al, AAF for England (2008) using the methods of Boffetta (2006) who estimated that the excess risk in the 1 to 19 g per day category to be half that reported for 25 g per day. The increase in relative risk of breast cancer per 10g per day was calculated as 7.1% by Hamajima et al.

**Supplementary Table 2.** Calculation of lifetime risk of cancer in alcohol abstaining never smokers

| **Cancer Type** | **ICD-10 code** | **Lifetime risk of cancer (R_0_)** | | **Alcohol attributable fractions (F_AA_)** | | **Tobacco attributable fractions (F_TA_)** | | **Adjusted alcohol attributable fractions (non-smokers)** | | **Adjusted tobacco attributable fractions (non-drinkers)** | | **Lifetime risk of cancer in abstaining never smoker (R_ANS_)** | |
| --- | --- | --- | --- | --- | --- | --- | --- | --- | --- | --- | --- | --- | --- |
|  |  |  | |  | |  | |  | |  | | R_ANS_ = R_0_(1–(F_AA_ + F_TA_)) | |
|  |  | Men | Women | Men | Women | Men | Women | Men | Women | Men | Women | Men | Women |
| Bladder | C67 | 2.56 | 0.94 | 0.000 | 0.000 | 0.38 | 0.34 | 0.000 | 0.000 | 0.38 | 0.34 | 1.59 | 0.62 |
| Colorectal* | C18, C20 | 7.18 | 5.43 | 0.170 | 0.120 | 0.07 | 0.10 | 0.090 | 0.064 | 0.01 | 0.01 | 6.47 | 5.02 |
| Breast | C50 | 0.12 | 12.90 | 0.000 | 0.130 | 0.00 | 0.00 | 0.000 | 0.130 | 0.00 | 0.00 | 0.12 | 11.22 |
| Cervix | C53 | 0.00 | 0.72 | 0.000 | 0.000 | 0.00 | 0.07 | 0.000 | 0.000 | 0.00 | 0.07 | 0.00 | 0.67 |
| Kidney | C64-C66,C68 | 1.81 | 1.11 | 0.000 | 0.000 | 0.29 | 0.15 | 0.000 | 0.000 | 0.29 | 0.15 | 1.29 | 0.94 |
| Larynx* | C32 | 0.40 | 0.40 | 0.370 | 0.240 | 0.79 | 0.79 | 0.196 | 0.127 | 0.09 | 0.09 | 0.28 | 0.31 |
| Leukaemia | C91-C95 | 1.52 | 1.04 | 0.000 | 0.000 | 0.19 | 0.06 | 0.000 | 0.000 | 0.19 | 0.06 | 1.23 | 0.98 |
| Liver* | C22 | 0.86 | 0.47 | 0.160 | 0.110 | 0.27 | 0.15 | 0.085 | 0.058 | 0.03 | 0.02 | 0.76 | 0.43 |
| Lung | C33-C34 | 7.63 | 5.77 | 0.000 | 0.000 | 0.85 | 0.80 | 0.000 | 0.000 | 0.85 | 0.80 | 1.14 | 1.15 |
| Oesophagus* | C15 | 1.80 | 0.91 | 0.600 | 0.480 | 0.63 | 0.71 | 0.318 | 0.254 | 0.08 | 0.09 | 1.09 | 0.60 |
| Oropharynx* | C00-C06,C09-C10,C12-C14 | 1.20 | 0.64 | 0.430 | 0.360 | 0.70 | 0.55 | 0.228 | 0.191 | 0.08 | 0.07 | 0.83 | 0.48 |
| Ovary | C56-C57 | 0.00 | 2.00 | 0.000 | 0.000 | 0.00 | 0.03 | 0.000 | 0.000 | 0.00 | 0.03 | 0.00 | 1.94 |
| Pancreas | C25 | 1.38 | 1.36 | 0.000 | 0.000 | 0.26 | 0.31 | 0.000 | 0.000 | 0.26 | 0.31 | 1.02 | 0.94 |
| Stomach | C16 | 1.57 | 0.84 | 0.000 | 0.000 | 0.26 | 0.15 | 0.000 | 0.000 | 0.26 | 0.15 | 1.16 | 0.71 |

*Cancers attributable to both alcohol and tobacco for which F_AA_ & F_TA_ were adjusted for the frequency of non-smokers and non-drinkers respectively

Adjusted F_AA_ for frequency of non-smoking drinkers: F_AA_ *0.53

Adjusted F_TA_ for frequency of non-alcohol consuming smokers: F_TA_ *0.12

**Supplementary Table 3.** Frequency of smoking and alcohol consumption in the UK, Health Survey for England data combined years 2011-14 (Public Health England n.d.)

| **Alcohol drinkers, n (%)** | | **Smokers, n (%)** | |
| --- | --- | --- | --- |
| Non-smoker | Smoker | Non-alcohol drinkers | Alcohol drinkers |
| 2025 (53.2) | 1108 (46.8) | 9059 (12.2) | 7974 (87.8) |

**Supplementary Table 4.** Calculation of absolute increase lifetime risk of cancer due to ten and 30 units of alcohol or ten and 30 cigarettes per week in non-smokers and non-drinkers respectively

|  |  | | **% Increase in relative risk of cancer**  **(RR-1)*100** | | | | | | | | **% Increase in absolute lifetime risk of cancer**  **R_ANS_*RR%** | | | | | | | |
| --- | --- | --- | --- | --- | --- | --- | --- | --- | --- | --- | --- | --- | --- | --- | --- | --- | --- | --- |
| Cancer Type | Lifetime risk of cancer in abstaining never smoker (R_ANS_) | | 10 units alcohol / week | | 30 units alcohol / week | | 10 cigarettes /  week | | 30 cigarettes /  week | | 10 units alcohol / week | | 30 units alcohol / week | | 10 cigarettes / week | | 30 cigarettes / week | |
|  |  | | RR% 10 units alcohol | | RR% 30 units alcohol | | RR% 10 cigarettes | | RR% 30 cigarettes | | R_ANS_*RR% 10 units alcohol | | R_ANS_*RR% 30 units alcohol | | R_ANS_*RR% 10 cigarettes | | R_ANS_*RR% 30 cigarettes | |
|  | Men | Women | Men | Women | Men | Women | Men | Women | Men | Women | Men | Women | Men | Women | Men | Women | Men | Women |
| Bladder | 1.59 | 0.62 | 0 | 0 | 0 | 0 | 16 | 3 | 54 | 8 | 0.00 | 0.00 | 0.00 | 0.00 | 0.25 | 0.02 | 0.86 | 0.05 |
| Colorectal | 6.47 | 5.02 | 4 | 4 | 7 | 7 | 8 | 8 | 24 | 24 | 0.26 | 0.20 | 0.45 | 0.35 | 0.49 | 0.38 | 1.57 | 1.22 |
| Breast | 0.12 | 11.22 | 0 | 7 | 0 | 21 | 0 | 1 | 0 | 3 | 0.00 | 0.79 | 0.00 | 2.36 | 0.00 | 0.10 | 0.00 | 0.29 |
| Cervix | 0.00 | 0.67 | 0 | 0 | 0 | 0 | 0 | 10 | 0 | 33 | 0.00 | 0.00 | 0.00 | 0.00 | 0.00 | 0.07 | 0.00 | 0.22 |
| Kidney | 1.29 | 0.94 | 0 | 0 | 0 | 0 | 14 | 0 | 49 | 0 | 0.00 | 0.00 | 0.00 | 0.00 | 0.18 | 0.00 | 0.64 | 0.00 |
| Larynx | 0.28 | 0.31 | 22 | 22 | 43 | 43 | 65 | 65 | 344 | 344 | 0.06 | 0.07 | 0.12 | 0.13 | 0.18 | 0.20 | 0.98 | 1.07 |
| Leukaemia | 1.23 | 0.98 | 0 | 0 | 0 | 0 | 7 | 7 | 23 | 23 | 0.00 | 0.00 | 0.00 | 0.00 | 0.09 | 0.07 | 0.28 | 0.22 |
| Liver | 0.76 | 0.43 | 10 | 10 | 19 | 19 | 12 | 12 | 41 | 41 | 0.08 | 0.04 | 0.14 | 0.08 | 0.09 | 0.05 | 0.31 | 0.18 |
| Lung | 1.14 | 1.15 | 0 | 0 | 0 | 0 | 10 | 12 | 33 | 41 | 0.00 | 0.00 | 0.00 | 0.00 | 0.11 | 0.14 | 0.37 | 0.47 |
| Oesophagus | 1.09 | 0.60 | 20 | 20 | 39 | 39 | 5 | 5 | 17 | 17 | 0.22 | 0.12 | 0.43 | 0.23 | 0.06 | 0.03 | 0.18 | 0.10 |
| Oropharynx | 0.83 | 0.48 | 43 | 43 | 86 | 86 | 13 | 13 | 45 | 45 | 0.36 | 0.20 | 0.71 | 0.41 | 0.11 | 0.06 | 0.38 | 0.22 |
| Ovary | 0.00 | 1.94 | 0 | 0 | 0 | 0 | 0 | 0 | 0 | 0 | 0.00 | 0.00 | 0.00 | 0.00 | 0.00 | 0.00 | 0.00 | 0.00 |
| Pancreas | 1.02 | 0.94 | 0 | 0 | 0 | 0 | 25 | 25 | 97 | 1 | 0.00 | 0.00 | 0.00 | 0.00 | 0.26 | 0.24 | 0.99 | 0.01 |
| Stomach | 1.16 | 0.71 | 0 | 0 | 0 | 0 | 20 | 20 | 73 | 73 | 0.00 | 0.00 | 0.00 | 0.00 | 0.23 | 0.14 | 0.85 | 0.52 |
| **Total** |  |  |  |  |  |  |  |  |  |  | **0.97** | **1.42** | **1.86** | **3.57** | **2.06** | **1.50** | **7.40** | **4.57** |

**Supplementary Table 5.** References and calculation of absolute increase lifetime risk of cancer due to ten and 30 cigarettes per week

| **Cancer Type** | **Metanalysis / Paper** | **Population** | **Total Subjects** | **Subjects for low levels smoking** | **Cig per day** | **Risk smoking approx. 5 cig per day (35 cig per week)** | | **Relative risk smoking**  **10 cig per week**  **exp(ln(RR_35cpw_)/3.5)** | | **Relative risk smoking**  **30 cig per week**  **exp(ln(RR_35cpw_)/1.7)** | |
| --- | --- | --- | --- | --- | --- | --- | --- | --- | --- | --- | --- |
|  |  |  |  |  |  | Men | Women | Men | Women | Men | Women |
| Bladder | Quirk et al. *Tobacco Induced Diseases*. 2:141 (2004) | America | **Case control study**  499 cases, 1922 controls | Men (30 cases, 149 control), Women (15 cases, 81 controls) | 1-10 | OR 1.66 (95% CI 0.99-2.6) | OR 1.1 (95% CI 0.6-2.2) | 1.16 | 1.03 | 1.54 | 1.08 |
| Colorectal* | Ordóñez-Mena et al. CHANCES Consortium.  *BMC Med.* 14: 62 (2016) | North America & Europe | **Metanalysis**  897,021 men & women | 29267 total, 514 cases | ≤ 9 | HR 1.29 (95% CI 1.17-1.43) | HR 1.29 (95% CI 1.17-1.43) | 1.08 | 1.08 | 1.24 | 1.24 |
| Breast* | Ordóñez-Mena et al. CHANCES Consortium.  *BMC Med.* 14: 62 (2016) | North America & Europe | **Metanalysis**  897021 men & women | 15852 total, 697 cases | ≤ 9 |  | HR 1.03 (95% CI 0.90-1.18) |  | 1.01 |  | 1.03 |
| Cervix | Roura et al. EPIC cohort. *Int J Cancer*. 15;135(2):453-66 (2014) | Europe | **Cohort Study**  308,036 women, 261 cases invasive cervical cancer | 34 cases | < 10 |  | HR 1.4 (95% CI 0.9-2.1) |  | 1.10 |  | 1.33 |
| Kidney | Hunt et al.  *Int J Cancer*. 10;114(1):101-08 (2005) | North America & Europe | **Metanalysis**  19 case-control studies (8,032 cases, 13,800 controls); 5 cohort studies (1,457,754 participants, 1,326 cases) |  | 1-9 | RR 1.60 (95% CI 1.21-2.12) | RR 0.98 (95% CI 0.71-1.35) | 1.14 | 0.99 | 1.49 | 0.98 |
| Larynx† | Hashibe et al. International Head & Neck Cancer Epidemiology Consortium. *J Natl Cancer Inst*. 16;99(10):777-89 (2007) | North America & Europe | **Metanalysis**  5 case-control studies - 10,244 head & neck cancer case subjects & 15,227 control subjects | 42 cases, 365 controls | 1-10 | OR 5.72 (95% CI 3.41-9.60) | OR 5.72 (95% CI 3.41-9.60) | 1.65 | 1.65 | 4.44 | 4.44 |
| Leukaemia | Fircanis et al. *Am J Hematol*. 89(8):E125-32 (2014) | North America & Europe | **Metanalysis**  23 studies: 2,139 cases, cohort 3,654,041 | 9 studies | < 10 | RR 1.27 (95% CI 1.03 - 1.56) | RR 1.27 (95% CI 1.03 - 1.56) | 1.07 | 1.07 | 1.23 | 1.23 |
| Liver‡ | Koh et al. Singapore Chinese Health Study. *Br J Cancer.* 105, 1430–1435 (2011) | Singapore & China | **Cohort Study**  63,257 men & women, 394 cases (low alcohol intake) | 46 cases | 1-12 | HR 1.49 (95% CI 1.06–2.09) | HR 1.49 (95% CI 1.06–2.09) | 1.12 | 1.12 | 1.41 | 1.41 |
| Lung | Gandini et al. *Int J Cancer*. 122(1):155-64 (2008) | International | **Metanalysis**  177 case-control studies, 75 cohorts & 2 nested case-control studies | 33 studies men; 25 studies women | 1-9 | RR 1.39 (95% CI 1.28-1.50) | RR 1.49 (95% CI 1.37-1.61) | 1.10 | 1.12 | 1.33 | 1.41 |
| Oesophagus* | Lagergren et al. *Int J Cancer*. 85(3):340-6 (2000) | Sweden | **Case control study**  189 cases, 820 controls |  | 1-9 | OR 1.2 (95% CI 0.7–2.2) | OR 1.2 (95% CI 0.7–2.2) | 1.05 | 1.05 | 1.17 | 1.17 |
| Oropharynx† | Hashibe et al. International Head & Neck Cancer Epidemiology Consortium. *J Natl Cancer Inst*. 16;99(10):777-89 (2007) | North America & Europe | **Metanalysis**  5 case-control studies - 10,244 head & neck cancer case subjects & 15,227 control subjects | 21 cases, 424 controls | 1-10 | OR 1.55 (95% CI 0.86-2.79) | OR 1.55 (95% CI 0.86-2.79) | 1.13 | 1.13 | 1.45 | 1.45 |
| Ovary | Faber et al. *Cancer Causes Control*. 24(5): 10.1007/s10552-013-0174-4 (2013) | North America & Europe | **Metanalysis**  11,972 invasive cases, 19,066 controls | 1,979 cases, 3,533 controls | 0-10 |  | OR 0.98 (95% CI 0.92-1.05) |  | 0.99 |  | 0.98 |
| Pancreas | Ordóñez-Mena et al. CHANCES Consortium.  *BMC Med.* 14: 62 (2016) | North America & Europe | **Metanalysis**  897,021 men & women | 29267 total, 156 cases | ≤ 9 | HR 2.21 (95% CI 1.83-2.67) | HR 2.21 (95% CI 1.83-2.67) | 1.25 | 1.25 | 1.97 | 1.97 |
| Stomach | Ordóñez-Mena et al. CHANCES Consortium.  *BMC Med.* 14: 62 (2016) | North America & Europe | **Metanalysis**  897,021 men & women | 29267 total, 87 cases | ≤ 9 | HR 1.90 (95% CI 1.46-2.47) | HR 1.90 (95% CI 1.46-2.47) | 1.20 | 1.20 | 1.73 | 1.73 |

* RR adjusted for alcohol intake

† RR excludes all drinkers

‡ RR excludes daily drinkers

**Supplementary Table 6.** Calculation of absolute increase lifetime risk of cancer due to ten units of alcohol or ten cigarettes per week in non-smokers and non-drinkers respectively following a sensitivity analysis

| **Cancer Type** | **Lifetime risk of cancer (R_0_)** | | **Alcohol Attributable Fraction (T_AA_)** | | **Tobacco Attributable Fraction (F_TA_)** | | **Lifetime risk of cancer in abstaining never smoker (R_ANS_)** | | **% increase in relative risk of cancer** | | | | **% Increase in absolute lifetime risk of cancer** | | | |
| --- | --- | --- | --- | --- | --- | --- | --- | --- | --- | --- | --- | --- | --- | --- | --- | --- |
|  |  | |  | |  | | R_ANS_ = R_0_(1–(F_AA_ + F_TA_)) | | RR% 10 units alcohol | | RR% 10 cigarettes | | R_ANS_*RR% 10 units alcohol | | R_ANS_*RR% 10 cigarettes | |
|  | Men | Women | Men | Women | Men | Women | Men | Women | Men | Women | Men | Women | Men | Women | Men | Women |
| Bladder | 2.56 | 0.94 | 0.00 | 0.00 | 0.38 | 0.34 | 1.59 | 0.62 | 0.00 | 0.00 | 15.58 | 2.76 | 0.00 | 0.00 | 0.25 | 0.02 |
| Colorectal* | 7.18 | 5.43 | 0.05 | 0.03 | 0.00 | 0.01 | 6.83 | 5.22 | 2.00 | 2.00 | 3.77 | 3.77 | 0.14 | 0.10 | 0.26 | 0.20 |
| Breast | 0.12 | 12.90 | 0.00 | 0.13 | 0.00 | 0.00 | 0.12 | 11.22 | 0.00 | 7.00 | 0.00 | 0.85 | 0.00 | 0.79 | 0.00 | 0.10 |
| Cervix | 0.00 | 0.72 | 0.00 | 0.00 | 0.00 | 0.07 | 0.00 | 0.67 | 0.00 | 0.00 | 0.00 | 10.09 | 0.00 | 0.00 | 0.00 | 0.07 |
| Kidney | 1.81 | 1.11 | 0.00 | 0.00 | 0.29 | 0.15 | 1.29 | 0.94 | 0.00 | 0.00 | 14.37 | 0.00 | 0.00 | 0.00 | 0.18 | 0.00 |
| Larynx* | 0.40 | 0.40 | 0.10 | 0.06 | 0.05 | 0.05 | 0.34 | 0.36 | 11.00 | 11.00 | 32.29 | 32.29 | 0.04 | 0.04 | 0.11 | 0.11 |
| Leukaemia | 1.52 | 1.04 | 0.00 | 0.00 | 0.19 | 0.06 | 1.23 | 0.98 | 0.00 | 0.00 | 7.07 | 7.07 | 0.00 | 0.00 | 0.09 | 0.07 |
| Liver* | 0.86 | 0.47 | 0.04 | 0.03 | 0.02 | 0.01 | 0.81 | 0.45 | 5.00 | 5.00 | 6.03 | 6.03 | 0.04 | 0.02 | 0.05 | 0.03 |
| Lung | 7.63 | 5.77 | 0.00 | 0.00 | 0.85 | 0.80 | 1.14 | 1.15 | 0.00 | 0.00 | 9.87 | 12.07 | 0.00 | 0.00 | 0.11 | 0.14 |
| Oesophagus* | 1.80 | 0.91 | 0.16 | 0.13 | 0.04 | 0.04 | 1.45 | 0.76 | 10.00 | 10.00 | 2.67 | 2.67 | 0.14 | 0.08 | 0.04 | 0.02 |
| Oropharynx* | 1.20 | 0.64 | 0.11 | 0.10 | 0.04 | 0.03 | 1.01 | 0.56 | 21.50 | 21.50 | 6.67 | 6.67 | 0.22 | 0.12 | 0.07 | 0.04 |
| Ovary | 0.00 | 2.00 | 0.00 | 0.00 | 0.00 | 0.03 | 0.00 | 1.94 | 0.00 | 0.00 | 0.00 | 0.00 | 0.00 | 0.00 | 0.00 | 0.00 |
| Pancreas | 1.38 | 1.36 | 0.00 | 0.00 | 0.26 | 0.31 | 1.02 | 0.94 | 0.00 | 0.00 | 25.43 | 25.43 | 0.00 | 0.00 | 0.26 | 0.24 |
| Stomach | 1.57 | 0.84 | 0.00 | 0.00 | 0.26 | 0.15 | 1.16 | 0.71 | 0.00 | 0.00 | 20.13 | 20.13 | 0.00 | 0.00 | 0.23 | 0.14 |
| **Total** |  |  |  |  |  |  |  |  |  |  |  |  | **0.58** | **1.15** | **1.65** | **1.17** |

**Supplementary Table 7.** Incidence and mortality data for alcohol-related cancers (CRUK data)

| **Cancer Type** | **Number of cases**  **(CRUK data 2015)** | **Number of deaths**  **(CRUK data 2014)** | **Deaths / Cases (%)** | **Survival (%)**  **(CRUK data)** | |
| --- | --- | --- | --- | --- | --- |
|  |  |  |  |  |  |
| Bladder | 10,171 | 5,369 | 52.8% | 50% | Survive for ≥ 10 years, 2010-11, England and Wales |
| Colorectal | 41,804 | 15,903 | 38.0% | 57% | Survive for ≥ 10 years, 2010-11, England and Wales |
| Breast | 55,122 | 11,433 | 20.7% | 78% | Survive for ≥ 10 years, 2010-11, England and Wales |
| Cervix | 3,126 | 890 | 28.5% | 63% | Survive for ≥ 10 years, 2010-11, England and Wales |
| Kidney | 12,547 | 4,421 | 35.2% | 50% | Survive for ≥ 10 years, 2010-11, England and Wales |
| Larynx | 12,061 | 839 | 7.0% | 19-59% | Survive for ≥ 10 years, 2009-13, England |
| Leukaemia | 9,900 | 4,584 | 46.3% | 46% | Survive for ≥ 10 years, 2009-13, England |
| Liver | 5,736 | 5,091 | 88.8% | 9% | Survive for ≥ 5 years, 2000-2007, England |
| Lung | 46,388 | 35,895 | 77.4% | 5% | Survive for ≥ 10 years, 2010-11, England and Wales |
| Oesophagus | 9,211 | 7,790 | 84.6% | 12% | Survive for ≥ 10 years, 2010-11, England and Wales |
| Oropharynx | 12,061 | 2,386 | 19.8% | 19-59% | Survive for ≥ 10 years, 2009-13, England |
| Ovary | 7,270 | 4,128 | 56.8% | 35% | Survive for ≥ 10 years, 2010-11, England and Wales |
| Pancreas | 9,921 | 8,817 | 88.9% | <1% | Survive for ≥ 10 years, 2010-11, England and Wales |
| Stomach | 6,740 | 4,576 | 67.9% | 15% | Survive for ≥ 10 years, 2010-11, England and Wales |
