# Supplementary material for: A comparison of gender-linked population cancer risks between alcohol and tobacco: how many cigarettes are there in a bottle of wine?
Source: BMC Public Health. 2019 Mar 28;19:316. doi: 10.1186/s12889-019-6576-9 (PMC6437970; doi:10.1186/s12889-019-6576-9)
Supplement: Supplementary file 2 — Contains a worked example of logarithmic transformation used to calculate RR of smoking 10 cigarettes per week. (DOCX 31 kb) [file 12889_2019_6576_MOESM2_ESM.docx]

**Appendix**

Worked example of logarithmic transformation used to calculate RR of smoking 10 cigarettes per week

p_0_ = probability of getting cancer when not smoking

p_1_ = probability of getting cancer when smoking 1 cigarette per week

𝑥 = number of cigarettes smoked per week

RR_1 =_ RR of smoking 1 cigarette per week, RR_35 =_ RR of smoking 35 cigarettes per week etc

ln(p_1_) = α + β𝑥 = α + β

ln(p_0_) = α + β𝑥 = α

ln(p_1_) - ln(p_0_) = β

ln(p_1_/p_0_) = β

(p_1_/p_0_) = exp(β) = RR_1_

ln(p_35_) = α + 35β (𝑥 = 35)

ln(p_0_) = α

ln(p_35_/p_0_) = 35β

ln(RR_35_) = 35β

ln(p_10_) = 10β

10β = 35β/3.5 = ln(RR_35_)/3.5

ln(RR_10_) = ln(RR_35_)/3.5

RR_10_ = exp((ln(RR_35_))/3.5)

Lung cancer example:

RR 1.39 for smoking 1-9 (approximately 5) cigarettes per day for men (35 cigarettes per week) (Gandini et al. *Int J Cancer*. 122(1):155-64 (2008))

RR_35_ = 1.39

RR_10_ = exp((ln(1.39))/3.5) = 1.10

RR_20_ = exp((ln(1.39))/1.75) = 1.21

RR_30_ = exp((ln(1.39))/1.17) = 1.33
